# Network Centrality as an Indicator for Pollinator Parasite Transmission via Flowers

**DOI:** 10.3390/insects11120872

**Published:** 2020-12-08

**Authors:** Niels Piot, Guy Smagghe, Ivan Meeus

**Affiliations:** Laboratory of Agrozoology, Department of Plants and Crops, Faculty of Bioscience Engineering, Ghent University Coupure Links 653, 9000 Ghent, Belgium; guy.smagghe@ugent.be (G.S.); ivan.meeus@ugent.be (I.M.)

**Keywords:** pollinator parasites, parasite transmission, plant–pollinator network, bee health

## Abstract

**Simple Summary:**

Understanding the transmission of disease is a key aspect to unravel the epidemiology of a disease. Multiple bee species face a global decline caused by an interplay of several factors, one of which is disease-causing parasites. Laboratory studies have identified that most of these parasites have an oral–fecal transmission route and that flowers may serve as a transmission spot between bee species. Within this study, we look if the transmission of parasites via flowers is actually occurring in the field under natural conditions. Furthermore, we look at plant–pollinator interactions, which can be represented as a network, and show that the centrality of a flower in the plant–pollinator network, weighted by visitation frequency, is a good predictor of the presence of parasites on the flower. In other words, we provide evidence to support the transmission mechanism via flowers in the field and show that flowers that are more central in the plant–pollinator network are most likely to contain parasites. Furthermore, we also explore the mechanism of external vectoring, where parasites hitchhike on the exterior of bees and are deposited on the flowers. This study further paves the path to epidemiological studies using the plant–pollinator network as a tool to assess the transmission of bee parasites.

**Abstract:**

Parasites are important actors within ecosystems. However, a key aspect to unraveling parasite epidemiology is understanding transmission. The bee pollinator community harbors several multihost parasites, which have been shown to be able to spread between species via flowers. Hence the plant–pollinator network can provide valuable information on the transmission of these parasites between species. Although several controlled experiments have shown that flowers function as a transmission hub for parasites, the link with the plant–pollinator network has rarely been addressed in the field. Here, one can hypothesize that the most central flowers in the network are more likely to enable parasite transmission between species. In this study, we test this hypothesis in three local plant–pollinator networks and show that the centrality of a plant in a weighted plant–pollinator network is a good predictor of the presence of multihost pollinator parasites on the plant’s flowers.

## 1. Introduction

Parasites are important players in shaping ecosystems [1]. Pollinators, like any other species, harbor a range of parasites, which include both macro- and microparasites [2,3]. Several of the microparasites are reported to be pathogenic and are found worldwide in domesticated pollinator species like honey bees [4,5]. Most of these microparasites found in honey bees are also present in wild bees [2,3,6,7]. Information on intraspecies transmission is scarce, let alone information on interspecies transmission. For social pollinators, e.g., honey bees and bumblebees, these parasites can readily spread within colonies due to close contact between individuals within the colony [8,9]. However, direct interspecies contact between different Anthophilia pollinators is rare, with only a few documented events of nectar robbing, in which one species enters the nest of another species [10]. Moreover, most of these multihost microparasites have an oral–fecal transmission route [2], enabling indirect transmission via flowers. Shared flowers can act as transmission hubs containing infective particles (i.e., parasitic cells, spores, or oocysts capable of infecting a naïve host) deposited by infected hosts, either due to defecation while foraging or through direct contact with the flower, transferring infective particles from the bee’s body to the flower. Conceptually, the role of flowers in the intraspecies transmission of parasites has been recognized [6,7,11,12,13,14,15]. However, only a few studies have been carried out in field conditions [6,7]. Understanding the role of the interaction between pollinators and flowers, i.e., the plant–pollinator network, in the spread of parasites is a key aspect when studying parasite epidemiology in the pollinator community as it provides information on the host–parasite transmission network [7]. In contrast to a previous study by Figueroa et al. (2020), using plant–pollinator networks to study parasite transmission, we mainly focused on the site of transmission, i.e., the flowers, to study the transmission mechanism instead of the host species. This is to further elucidate the role of the plant–pollinator network and transmission mechanisms leading to the contamination of flowers with parasites.

Flower visitation rates of potential hosts will most likely be an important parameter to describe the transmission via flowers. Here, one might expect that flowers that have a high visitation rate by different pollinators and the shortest total distance to all other nodes in the network are more likely to contain parasites. These flowers can be regarded as the most central flowers in a network weighted with visitation rates. Following this rationale, we pose the hypothesis that central flowers in a plant–pollinator network have a higher likelihood of carrying parasites. If the hypothesis is valid, it also further underlines the usefulness of plant–pollinator network characteristics in parasite transmission in a natural environment. We tested this hypothesis in three local plant–pollinator networks, mostly dominated by *Bombus* spp., and looked at two groups of microparasites typically associated with bee pollinators, i.e., Trypanosomatidae and Neogregarinorida. Both have an oral–fecal transmission route and have a broad host range within the bee pollinator community [3,16]. To examine if external vectoring is a possible transmission route, we investigated the presence of microparasites on the exterior of the bees and looked if there was a link with parasite presence in the bees. Furthermore, external parasite loads were quantified to see if they could theoretically lead to an infection.

## 2. Materials and Methods

### 2.1. Study Area

The study was carried out at three separate study sites: two study sites were in a rural area and a third study site was located in an urban area (Figure S1). All sampling and monitoring were performed at the beginning of June. Study Site 1 was sampled in 2016 in Zelzate, Belgium (N 51.2070, E 3.7913) in a road ditch next to a wheat field. The study site had a total size of 50 by 1.5 m. Study Site 2 was sampled in 2017 in Zelzate, Belgium (N 51.2052, E 3.8379) in a road ditch next to two wheat fields. The site had a size of 70 by 2.5 m. Study Site 3 was sampled in 2017 in Ghent, Belgium (N 51.0536, E 3.7060). Located in an urban area, the study site had a total size of 40 by 1.5 m. All flowers present in each plot were counted and determined to species level (Table S1). A transect walk was performed by walking along the whole length of the study site twice; all flower-visiting pollinators were recorded through visual inspection to give an estimate on their presence and activity (Table S2). This allowed us to set up a monitoring plan to measure visitation data per plant species generated from camera recording (see Section 2.2 for network construction). Most flower-visiting pollinators were hymenopterans. However, in Study Site 1, there was also a high activity of dipteran species.

The network in Study Site 1 included four flower species (i.e., *Symphytum officinale*, *Lamium album*, *Aegopodium podagraria*, and *Geranium pusillum*). In Study Site 2, the network also included four flower species (i.e., *Symphytum officinale*, *Lamium album, Aegopodium podagraria*, and *Rubus fruticosus*). In Study Site 3, the network consisted of five flower species (*Malva neglecta*, *Scabiosa columbaria*, *Papaver rhoeas*, *Salvia pratensis,* and *Leucanthemum vulgare*). All plants were native to Belgium (see Figure S2 for further information on plant species) [17,18].

### 2.2. Network Construction

**Figure 1:**
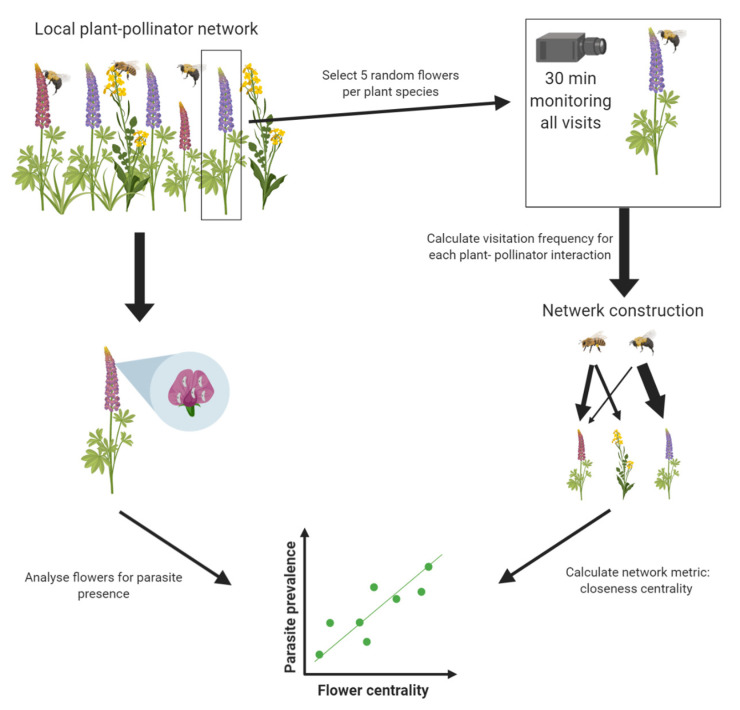
Schematic overview of the network construction and relationship between flower centrality and parasite prevalence. In three local plant–pollinator networks, 5 random flowers of each plant species present at the site were recorded using a camera. This data was used to construct the weighted plant–pollinator networks, where we calculated the visitation frequency for each plant–pollinator interaction (see Section 2.2 on network construction for full details). From the resulting networks, the centrality of each plant species was calculated. We analyzed the parasite prevalence on the flowers of each plant species and looked at the relationship between parasite prevalence on the flowers and flower centrality using a generalized linear mixed model.

At each site, plant–pollinator networks were constructed using visitation data generated from camera recordings (see Figure 1 for a schematic overview of network construction). At each site, five random flowers of different individual plants were selected for each plant species present at the site. Each selected flower was recorded for 30 min (±42 s). All recordings for one site were performed on the same day. Recordings were performed using two cameras (Nikon COOLPIX P510, Minato City, Tokyo, Japan and Panasonic DMC-TZ35, Kadoma, Osaka, Japan) on a tripod positioned in front or above each selected flower to provide a full and focused view of the recorded flowers. The video recording of each flower was analyzed afterward using Windows Media Player 12 (Windows 10, Microsoft, Redmond, WA, USA). For each flower, the number of visits was counted for each pollinator species. An average visitation frequency (visits/min) was calculated for each flower species–pollinator species interaction using the following equation:

(1) $$\frac{\sum_{n=1}^{5}\frac{number\;of\;visits\;of\;flower\;n\;by\;the\;pollinator\;species}{recording\;time}}{5}$$

**Figure 2:**
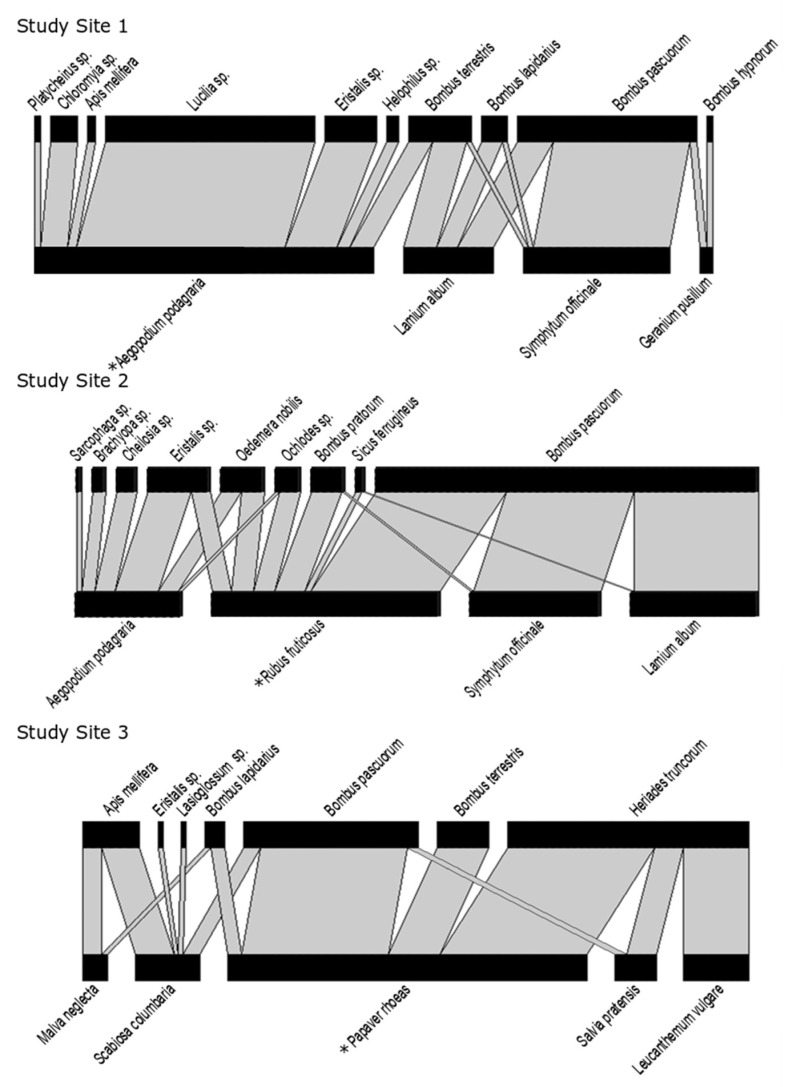
Visualized weighted plant–pollinator networks for each study site; the width of the grey bars corresponds to the average visitation frequency. The most central flower (based on normalized closeness) is indicated with * for each network.

Each network was weighted based on the average visitation frequency calculated for each plant–pollinator interaction, as described above. This resulted in three weighted bipartite plant–pollinator networks, one for each study site (see Figure 2). For each network, the properties were calculated with the tnet package [19] in R [20]. As a measure of centrality in the plant–pollinator network, we used closeness, which is often used to approximate centrality in a network [19,21,22]. The closeness centrality can be defined as the inverse of farness (farness is the sum of the distances to all other nodes in the network) [19]. The closeness of each node was normalized by dividing it by the total number of nodes in the network minus one, resulting in normalized closeness (a value between 0 and 1). By using a parameter α of 0.5 in the tnet package, both the number of ties (i.e., edges) as well as the weights of the ties (determined by the average visitation frequency) were scored as positive in the calculation of normalized closeness [19]. The normalized closeness of each plant species was used as a fixed factor for Model 2 (see below).

### 2.3. Sampling and Parasite Analysis

To determine parasite presence and load on the flowers in the network, the flowers of each species were cut off at the pedicel, just below the sepals, and put in separate tubes at the end of the experiment (i.e., after all the recordings needed to construct the network were finished). On average, 20 flowers per species were sampled, with a minimum of 10 flowers for *G. pusillum*. For details on the sampling amount of each flower species at each site, see Table S3. To get an estimate on parasite prevalence in the bee pollinators present at each site and to test if the parasites were present on the exterior of the bees, a minimum of 10 bees of the most abundant bee species was sampled at each location (see Table S6 for detailed sampling amounts). Each bee was stored in a separate container for transport and sacrificed by freeze-killing in the lab. Bees and flowers were stored at −80 °C until further use.

Flowers were washed in 1 mL of RLT buffer (Qiagen, Venlo, The Netherlands) supplemented with 1% β-mercapto-ethanol. Washing was done by shaking in the Qiagen TissueLyser II for 2 min at 30 Hz, followed by 2 min at 20 Hz with 0.3 g of silica beads (0.1 mm; BioSpec Products, Bartlesville, OK, USA). After washing, the flower was removed, and the homogenate was centrifuged for 2 min at 2000× *g*; then, 200 µL of supernatant was used for DNA extraction.

If bees indeed transfer parasites via contact, one would expect the parasites to be present on the exterior of the bees. To test this, bees were washed with 1 mL of RLT buffer supplemented with 1% β-mercapto-ethanol by vortexing for 1 min. Prior to washing, the hind legs of the bees were removed for *Bombus* spp. to prevent contamination with corbicular pollen. For *Heriades truncorum*, pollen present on the abdomen was removed with a small paintbrush. We note here that the release of fecal droplets by the bee during vortexing cannot be excluded. Then, 200 µL of the wash solution was used for DNA extraction.

To detect the presence of parasites within the bees, the abdomen of the washed bees was cut off and homogenized in 800 µL of RLT buffer supplemented with 1% β-mercapto-ethanol for *Bombus* spp. or 400 µL of RLT buffer supplemented with 1% β-mercapto-ethanol for *Heriades truncorum*. Homogenization was performed with the Qiagen TissueLyser II for 2 min at 30 Hz, followed by 2 min at 20 Hz with one stainless steel bead of 5 mm and three stainless steel beads of 3 mm. The homogenate was centrifuged for 2 min at 2000× *g*, and 200 µL of supernatant was used for DNA extraction. DNA extraction was performed with the Invisorb Spin Tissue Mini Kit (Stratec Biomedical, Birkenfeld, Germany). Then, 200 µL homogenate, as described above, was added to 400 µL of lysis buffer G and 40 µL of proteinase S. Further extraction steps proceeded according to the manufacturer’s instructions (Protocol 1). DNA was stored at −20 °C until further use. Detection of Trypanosomatidae and Neogregarinorida was done with primers described by Meeus et al. (2009). All PCRs included positive, negative, and no-template controls. Several positive samples were sent for Sanger sequencing to confirm the identity of the detected parasites (LGC Genomics, Berlin, Germany). All parasites detected with the primers described by Meeus et al. (2009) were either *Crithidia* spp. (parasites belonging to the Trypanosomatidae family) or *Apicystis bombi* (a parasite belonging to the Neogregarinorida family).

### 2.4. Parasite Quantification

To enable the infection of a naïve host upon visitation of a parasite-contaminated flower, the number of infective particles present on the flower should surpass the minimal amount needed to induce an infection in a host. To quantify the parasite load of the detected parasites, the CFX 96 Touch Real-Time PCR Detection System (Bio-Rad, Hercules, CA, USA) was used. Each well contained 8 µL of the sample, 10 µL of GoTaq qPCR master mix (Promega, Madison, WI, USA), 1 µL (10 µM) of forward primer, and 1 µL (10 µM) of reverse primer [23]. All samples were run in duplicate, and each plate included negative controls. Absolute quantification of the parasite load on the flowers and exterior of the bees was done based on the dilution curves of DNA extracts from a sample with a known number of infective particles. To generate a standard curve for quantification of Trypanosomatidae (E = 87.2%; R^2^ = 0.991), we used an in vitro lab culture of *Crithidia mellificae*. To generate a standard curve for quantification of Neogregarinorida, we used isolated oocysts of *A. bombi* (E = 91.4%; R^2^ = 0.992). *A. bombi* was isolated from a highly infected wild-caught *B. pascuorum* worker (this bee was caught in Ghent, Belgium, but was not part of the networks used in this study); fat body and gut tissue were ground in PBS solution. Oocysts of *A. bombi* were purified by 90 min centrifugation at 3450× *g* over a gradient consisting of 1 mL of each layer of 30%, 50%, 70%, and 90% Percoll (Sigma-Aldrich, Overijse, Belgium). The interface between the 70% and 90% Percoll was collected and diluted with 2 mL of PBS and centrifuged at 870× *g* for 15 min; this was repeated 3 times to replace the Percoll with PBS in order to obtain purified *A. bombi* oocysts in PBS solution.

A subset of both the *C. mellificae* cells (from the in vitro culture) and the *A. bombi* oocysts (isolated from a *B. pascuorum* worker, as described above) was taken, and the cells/oocysts were counted with a hemocytometer (Bürker chamber) before DNA extraction. By using a standard curve based on an extraction of a known number of infective particles, we compensate for DNA extraction efficiency. However, we cannot compensate for the efficiency of the wash steps of both the flowers and the bees. Therefore, the obtained number of infective particles is indicative of the minimal amount present on both the flowers and the exterior of the bees. The parasite load in the bumblebees was quantified relatively (relative to the lowest parasite load) since absolute quantification here is not appropriate. As the host replication stages of the parasites may be present for Neogregarinorida [24], this could result in an overestimation of the number of parasites present.

### 2.5. Statistical Analysis

To explain the parasite presence on the flowers, we compared two generalized linear mixed models (GLMMs) using the lme4 package [25]. For these models, the presence or absence of parasites (i.e., either Trypanosomatidae or Neogregarinorida) served as a binomial response variable for which the link function log of the odds ratio (logit) was used. Site and flower species served as random variables, where flower species was nested within the site. In the first model (M1), we used the average visitation frequency (of all pollinators combined) per plant species as a fixed factor (Equation (2)).

(2) $$\frac{\sum_{n=1}^{5}\frac{number\;of\;visits\;by\;all\;pollinators\;on\;flower\;n}{record\;time}}{5}$$

In the second model (M2), we used the natural logarithm of normalized closeness as a fixed factor (see Section 2.2 on network construction for details) as this measure is most often used to approximate centrality in a network [19,21,22].

The relationship between the detection of microparasites in the bees and the presence of microparasites of the same species on the exterior of the bees was tested using Pearson’s chi-squared test with Yates’ correction. The relationship between the relative quantity of the detected parasites (i.e., parasite load) in the bees and the presence of microparasites of the same species on the exterior of the bees was tested using ANOVA, where the relative quantity of the parasite was made relative to the individual with the lowest parasite load.

## 3. Results

### 3.1. Network

A total of 32 h and 30 min of observation data across the three sites resulted in a total of 414 observed interactions. In Study Site 1, 169 interactions were observed; 158 and 87 interactions were observed in Study Sites 2 and 3, respectively. The observed plant–pollinator networks are visualized in Figure 2. With 195 observed interactions across all sites, *Bombus pascuorum* was the most observed species across study sites, accounting for 47% of all observed interactions. In Study Site 1, the most central flower (based on normalized closeness centrality) in the plant–pollinator network was *Aegopodium podagraria*; in Study Sites 2 and 3, these were *Rubus fruticosus* and *Papaver rhoeas*, respectively.

### 3.2. Parasite Prevalence and Flower Centrality

We investigated the presence of two groups of microparasites, i.e., Trypanosomatidae and Neogregarinorida. In general, we found that 31.1% of the 273 analyzed flowers contained one of the investigated microparasites. Trypanosomatidae were more prevalent than Neogregarinorida on the flowers, with a prevalence of 26% and 7.3%, respectively. Here, we tested our hypothesis that the centrality of a flower species in a pollination network is a good predictor for the presence of microparasites, i.e., the more central the flower in the network, the higher the likelihood that the flower will contain microparasites.

To ensure that the presence of microparasites is not merely a reflection of the visitation of the flowers, we compared two models to explain parasite prevalence on the flowers. In the first model (M1), we used the average visitation frequency per plant species (Equation (2)), while in the second model (M2), we used the centrality of the plant within the plant–pollinator network, measured as normalized closeness, as the fixed factor. Both factors had a weak correlation (r = 0.18, df = 271, *p* = 0.003, Pearson’s correlation).

**Figure 3:**
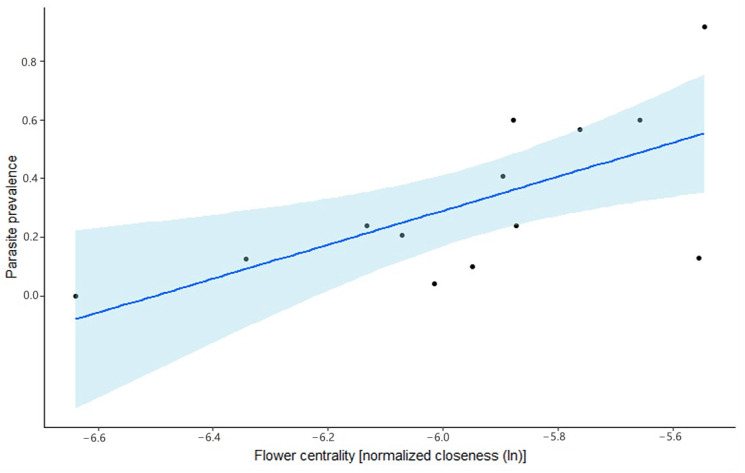
Results of the generalized linear mixed Model 3 (GLMM 3): interaction effect of flower centrality (ln-transformed normalized closeness; *x*-axis) and parasite prevalence (*y*-axis), i.e., Neogregarinorida and Trypanosomatidae taken together (presence/absence data). Parasite prevalence and centrality are plotted (black dots) for each analyzed plant species in each study site. Shaded blue shows the 95% confidence interval.

M2 emerged as the best model, with the lowest Akaike information criterion (see Table 1). The metric centrality, derived from the weighted plant–pollinator network, is hence more informative in predicting the likelihood of parasite presence on the flowers compared to visitation frequency alone. In M2, we also provide evidence for our hypothesis, as here, the more central a flower species in the pollination network, the more likely it is to contain microparasites (see Figure 3). When the parasites were analyzed separately, the effect was only significant for the Trypanosomatidae (see Table S7).

### 3.3. Parasite Quantification

The number of Neogregarinorida parasites found on the flowers and the exterior of the bees ranged from 8 to 7186 and 9 to 138, respectively. For Trypanosomatidae, the number of parasites on the flowers and the exterior of the bees ranged from 6 to 4701 and 5 to 1436, respectively (see Figures S3–S6 and the accompanying Tables S4 and S5).

On the outside of the bees, we could detect the presence of Neogregarinorida and Trypanosomatidae on 7.7% and 36.9% of the screened bees, respectively. Parasite prevalence in the screened bees, i.e., *Bombus pascuorum*, *Bombus lapidarius*, *Bombus terrestris,* and *Heriades truncorum* was relatively high: 41.5% and 63.1% of the screened bees harbored *A. bombi* (belonging to the Neogregarinorida family; confirmed by Sanger sequencing) and *C. bombi* (belonging to the Trypanosomatidae family; confirmed by Sanger sequencing), respectively (see Table S6 for an overview and Figures S7–S8 for the relative quantification). However, none of the *H. truncorum* bees harbored Trypanosomatidae, although these parasites were found on the exterior of some of these bees. Codetection of *C. bombi* and *A. bombi* occurred in 18.5% of the screened bees. Out of all bees analyzed, 17% carried parasites on their exterior and did not harbor the parasite themselves. This was most frequently found for Trypanosomatidae, where 10 bees were found to have Trypanosomatidae parasites on their exterior, yet the bees did not harbor this parasite; most of these bees were *H. truncuorum* (6 out of 10). We found only one case where Neogregarinorida were present on the exterior and were not present within the bee. There was no relationship between the presence of parasites in the bee and the presence of parasites on the outside of the bee, neither for Neogregarinorida (χ^2^ = 1.601, *p* = 0.206) nor for Trypanosomatidae (χ^2^ = 0.116, *p* = 0.734). However, the relative quantity of detected parasites within the bee was strongly linked with the presence of parasites on the outside of the bee for Neogregarinorida (F_1,63_ = 14.54, *p* < 0.001) and for Trypanosomatidae (F_1,63_ = 4.273, *p* = 0.043), where a higher relative parasite quantity within the bee was linked with a higher likelihood of an external presence of the parasites.

## 4. Discussion

Unraveling the transmission process is an important facet of disease epidemiology. Nevertheless, under natural conditions, it is often difficult to determine the exact mechanisms of transmission, especially when dealing with multihost parasites [26]. In this study, we identified flowers as a potential transmission hub for multihost parasites, i.e., Neogregarinorida and Trypanosomatidae, in a pollinator community mostly dominated by bee species. The transmission process of parasites can be broken down into different steps: (i) infectious particles are presented by an infected host, (ii) infectious particles are dispersed between healthy and infected hosts, and (iii) infectious particles are taken up by a naïve host, which becomes infected [26]. Screening of the flowers in our network showed the presence of both Trypanosomatidae and Neogregarinorida on different flower species. These findings are in line with the dispersion of infective particles by an infected host (i.e., step two in the transmission process) via flowers. This route has been put forward in several studies under controlled circumstances [11,13,27]. Recently two studies have also found the presence of parasites on flowers in field conditions and provide valuable insights into the role of plant–pollinator networks in the transmission of parasites [6,7]. Figueroa et al. (2020) showed that network characteristics, such as degree (i.e., the diet breath) of the most dominant pollinator, as well as the general network connectance, are good predictors of parasite prevalence in the pollinator community. Although this study provides interesting results, the construction of their networks was done by observations over several months, which may overlook detailed processes of transmission as the plant–pollinator networks change over time [28]. This turnover will likely affect parasite transmission. Graystock et al. (2020) found that parasite prevalence on flowers changes with the seasons. Within this study, we performed extensive monitoring (average observed interactions per network was 138 one-day observations) in comparison to Figueroa et al. (2020; average observed interactions per network was 267 (several days of monitoring spread across months)) to elucidate the transmission process and its link to the plant–pollinator network in more detail. Furthermore, by focusing on the flowers, i.e., the site of transmission, to elucidate the role of the plant–pollinator network in parasite transmission, we overcome several factors that may obscure the findings when one focuses on the host species. One clear example here is the presence of social pollinators, such as honey bees or bumblebees, which may have acquired the parasite infection from close contact within the nest [29,30] rather than via flowers; this may bias parasite prevalence in the analyzed hosts if these social bees are abundant.

Besides the detection of parasites on flowers, which is in line with previous reports [6,7], we also show that the parasite loads detected on the flowers surpass the minimal amount needed to induce an infection under laboratory conditions, based on the current literature [27,31].

The presentation of infective particles by an infected host on the flowers (i.e., step one of the transmission process) can occur via feces as both Neogregarinorida and Trypanosomatidae occurring in bee species have an oral–fecal transmission, where feces can contain high loads of infective particles [32]. Graystock et al. (2013) showed that feeding off the feces containing *A. bombi* (as low as ca. 40 oocysts) can result in an infection. Infection studies with feces have also been performed in several studies for *C. bombi*, a bee-infecting trypanosomatid [32,33].

External vectoring, where a pollinator deposits infective particles that are sticking to its body onto a flower, could also result in the contamination of flowers. We here evaluated the likelihood of this mechanism by quantifying the parasite loads on the exterior of bees. We are aware that the detection and quantification of infective particles are not direct proof of the existence of this mechanism, yet it provides information on the potential of this transmission mechanism.

We here show that bees can indeed carry infectious particles on their exterior. As one can expect, we found that bees that had a high internal parasite load were more likely to have parasites on their exterior. However, we also found that parasites can be present on the exterior without the bee harboring the parasites internally. Quantification of the infective particles on the exterior showed that both *C. bombi* and *A. bombi* exceeded the minimal infection amount currently reported in the literature (*vide infra*). The absence of a link between the detection of parasites within the bee and the presence of infective particles on their exterior, together with the detected quantity on the bee’s exterior, strongly suggest that external vectoring is indeed a possible route for the dispersal of infective particles. Finally, for an infection to occur, infective particles must be taken up by a naïve host (step three in the transmission process). The number of infective particles taken up must surpass the minimal amount needed to establish an infection. Quantification of the infective particles on the flowers of both Trypanosomatidae and Neogregarinorida showed that both exceeded the minimal infection dose currently reported in the literature to induce an infection. The Neogregarinorida loads found on the flowers were up to 100-fold higher than the minimal infection dose needed, i.e., 40 oocysts, according to the current literature [31]. For the trypanosome *C. bombi*, the minimal infection dose found in the literature is circa 1000 infective cells [27], which is quite high compared to the neogregarine *A. bombi*. Either this dose is indeed the minimal possible infection dose or lower doses are not tested, as most studies want to ensure infection in their experiments. Nonetheless, we found Trypanosomatidae loads on the flowers that were in the same order of magnitude as the minimal infection dose published in the literature. However, we do not know how many infective particles are taken up from a flower by a visiting naïve host, as this is dependent on the flower part where the infective particles are encountered, which is, in turn, dependent on the plant species and the pollinator species visiting the plant [15]. Hence, the quantification of infective particles merely shows that the parasite quantities present on the flower are high enough to potentially cause an infection if ingested. Within this study, we stated the hypothesis that if transmission indeed occurs via flowers, then central flowers in the plant–pollinator network have a higher likelihood of carrying infective particles. We compared two factors to explain parasite prevalence on the flowers and showed that using the flower’s closeness centrality (obtained from a weighted plant–pollinator network) is more informative than using the visitation frequency of the flower. These results provide evidence to support our hypothesis and further underline current insights into the usefulness of plant–pollinator networks in epidemiological studies on pollinators. This study, together with the recent study of Figueroa et al. (2020), provides the first steps into a thorough understanding of the role of the plant–pollinator network in parasite transmission. We, therefore, argue that future research should aim at looking at both the role of the host species (as done by Figueroa et al. (2020)) and the role of the flowers (this study) in intense network monitoring (i.e., visitation weighted network construction based upon data collected on a single day) at different times to provide more detail on the effect of the temporal dynamics of plant–pollinator networks on parasite transmission and the role of the hosts and flowers herein.

## 5. Conclusions

In this study, we provide further evidence to support in-field transmission of multihost parasites via flowers and show that the quantities detected on the flowers in the field are within the range to induce an infection if ingested. Furthermore, we show that a network metric, i.e., closeness centrality from the plant–pollinator network, can be used as a good proxy for the likelihood of a flower to contain parasites. Lastly, we showed that the quantities of parasites detected on the exterior of bees is, in theory, enough for external vectoring of parasites between flowers to occur in the field. This study provides insights to further elucidate the transmission mechanisms of bee parasites in the pollinator community using the plant–pollinator network.

## Figures and Tables

**Table 1:** Comparison of the null model and two generalized linear mixed models with different fixed factors to explain parasite prevalence, i.e., Neogregarinorida and Trypanosomatidae taken together (binomial logit distributed) for all model study sites served as a random effect, and flower species was nested within the study site. Models were compared based upon the Akaike information criterion. * ΔAIC with regard to the null model (i.e., model that included the same random effect as the other models, yet no fixed factor).

Model	Factor	β	df	χ^2^	*p*-Value	ΔAIC *
M1	Visitation frequency	−0.494	4	0.051	0.822	+2.0
M2	Normalized Closeness	3.840	4	8.382	0.0038	−5.4

## Supplementary Materials

The following are available online at https://www.mdpi.com/2075-4450/11/12/872/s1, Figure S1: Pictures of the different study sites, Figure S2: Pictures and descriptions of each plant species used in this study, Figure S3: Dot plot of the quantification of Crithidia spp. on the flowers, Figure S4: Dot plot of the quantification of *A. bombi* on the flowers, Figure S5: Dot plot of the quantification of *Crithidia* spp. found on the exterior of the bees, Figure S6: Dot plot of the quantification of *A. bombi* found on the exterior of the bees, Figure S7: Boxplot of the relative quantification of *Crithidia* spp. found in the bees per study site, Figure S8: Boxplot of the relative quantification of *A. bombi* found in the bees per study site, Table S1: Flower species presence and abundance per species at the study sites, Table S2: Pollinator counts from the performed transects at each study site, Table S3: An overview of the number of sampled flowers for parasite analysis at each study site, Table S4: An overview of the quantification of *Crithidia* spp. of the positive flowers per study site, Table S5: An overview of the quantification of *Apicystis bombi* of the positive flowers per study site, Table S6: An overview of the number of sampled bee species at each study site and the number of *C. bombi-* and *A. bombi*-infected individuals, Table S7: Results of the GLMM for the separate parasites.

## References

[1] Hudson P.J., Dobson A.P., Lafferty K.D. (2006). Is a healthy ecosystem one that is rich in parasites?. Trends Ecol. Evol..

[2] Goulson D., Hughes W.O. (2015). Mitigating the anthropogenic spread of bee parasites to protect wild pollinators. Biol. Conserv..

[3] Ravoet J., de Smet L., Meeus I., Smagghe G., Wenseleers T., de Graaf D.C. (2014). Widespread occurrence of honey bee pathogens in solitary bees. J. Invertebr. Pathol..

[4] Schwarz R.S., Bauchan G.R., Murphy C.A., Ravoet J., de Graaf D.C., Evans J.D. (2015). Characterization of two Species of Trypanosomatidae from the honey bee *Apis mellifera*: *Crithidia mellificae* Langridge and McGhee, and Lotmaria passim n. gen., n. sp.. J. Eukaryot. Microbiol..

[5] Graystock P., Meeus I., Smagghe G., Goulson D., Hughes W.O.H. (2016). The effects of single and mixed infections of *Apicystis bombi* and deformed wing virus in *Bombus terrestris*. Parasitology.

[6] Graystock P., Ng W.H., Parks K., Tripodi A.D., Muñiz P.A., Fersch A.A., Myers C.R., McFrederick Q.S., McArt S.H. (2020). Dominant bee species and floral abundance drive parasite temporal dynamics in plant-pollinator communities. Nat. Ecol. Evol..

[7] Figueroa L.L., Grab H., Ng W.H., Myers C.R., Graystock P., McFrederick Q.S., McArt S.H. (2020). Landscape simplification shapes pathogen prevalence in plant-pollinator networks. Ecol. Lett..

[8] Otti O., Schmid-Hempel P. (2008). A field experiment on the effect of *Nosema bombi* in colonies of the bumblebee *Bombus terrestris*. Ecol. Entomol..

[9] Erler S., Popp M., Wolf S., Lattorff H.M.G. (2012). Sex, horizontal transmission, and multiple hosts prevent local adaptation of *Crithidia bombi*, a parasite of bumblebees (Bombus spp.). Ecol. Evol..

[10] Andrews C. (1969). The Lives of Wasps and Bees.

[11] Graystock P., Goulson D., Hughes W.O.H. (2015). Parasites in bloom: Flowers aid dispersal and transmission of pollinator parasites within and between bee species. Proc. R. Soc. B Biol. Sci..

[12] Singh R., Levitt A.L., Rajotte E.G., Holmes E.C., Ostiguy N., Lipkin W.I., dePamphilis C.W., Toth A.L., Cox-Foster D.L. (2010). RNA viruses in Hymenopteran pollinators: Evidence of inter-taxa virus transmission via pollen and potential impact on non-Apis Hymenopteran species. PLoS ONE.

[13] Durrer S., Schmid-Hempel P. (1994). Shared use of flowers leads to horizontal pathogen transmission. Proc. R. Soc. B Biol. Sci..

[14] Alger S.A., Burnham P.A., Brody A.K. (2019). Flowers as viral hot spots: Honey bees (*Apis mellifera*) unevenly deposit viruses across plant species. PLoS ONE.

[15] Figueroa L.L., Blinder M., Grincavitch C., Jelinek A., Mann E.K., Merva L.A., Metz L.E., Zhao A.Y., Irwin R.E., McArt S.H. (2019). Bee pathogen transmission dynamics: Deposition, persistence and acquisition on flowers. Proc. R. Soc. B Biol. Sci..

[16] Tian T., Piot N., Meeus I., Smagghe G. (2018). Infection with the multi-host micro-parasite *Apicystis bombi* (Apicomplexa: Neogregarinorida) decreases survival of the solitary bee *Osmia bicornis*. J. Invertebr. Pathol..

[17] Eggeltje H., Lid D.T. (2015). Veldgids Nederlandse Flora.

[18] van Landuyt W., Vanhecke L., Brosens D. (2012). Florabank1: A grid-based database on vascular plant distribution in the northern part of Belgium (Flanders and the Brussels Capital region). PhytoKeys.

[19] Opsahl T., Agneessens F., Skvoretz J. (2010). Node centrality in weighted networks: Generalizing degree and shortest paths. Soc. Netw..

[20] R Core Team (2018). R: A Language and Environment for Statistical Computing.

[21] Freeman L.C. (1978). Centrality in social networks conceptual clarification. Soc. Netw..

[22] González A.M.M., Dalsgaard B., Olesen J.M. (2010). Centrality measures and the importance of generalist species in pollination networks. Ecol. Complex..

[23] Meeus I., de Graaf D.C., Jans K., Smagghe G. (2009). Multiplex PCR detection of slowly-evolving trypanosomatids and neogregarines in bumblebees using broad-range primers. J. Appl. Microbiol..

[24] Lipa J.J., Triggiani O. (1996). *Apicystis gen nov* and *Apicystis bombi* (Liu, Macfarlane & Pengelly) comb nov (Protozoa: *Neogregarinida*), a cosmopolitan parasite of *Bombus* and *Apis* (Hymenoptera: *Apidae*). Apidologie.

[25] Bates D., Mächler M., Bolker B., Walker S. (2015). Fitting Linear Mixed-Effects Models Using lme4. J. Stat. Softw..

[26] Antonovics J. (2017). Transmission dynamics: Critical questions and challenges. Philos. Trans. R. Soc. B Biol. Sci..

[27] Otterstatter M.C., Thomson J.D. (2008). Does pathogen spillover from commercially reared bumble bees threaten wild pollinators?. PLoS ONE.

[28] CaraDonna P.J., Petry W.K., Brennan R.M., Cunningham J.L., Bronstein J.L., Waser N.M., Sanders N.J. (2017). Interaction rewiring and the rapid turnover of plant-pollinator networks. Ecol. Lett..

[29] Folly A.J., Koch H., Stevenson P.C., Brown M.J.F. (2017). Larvae act as a transient transmission hub for the prevalent bumblebee parasite *Crithidia bombi*. J. Invertebr. Pathol..

[30] Otterstatter M.C., Thomson J.D. (2007). Contact networks and transmission of an intestinal pathogen in bumble bee (*Bombus impatiens*) colonies. Oecologia.

[31] Graystock P., Yates K., Evison S., Darvill B., Goulson D., Hughes W.O.H. (2013). The Trojan hives: Pollinator pathogens, imported and distributed in bumblebee colonies. J. Appl. Ecol..

[32] Otterstatter M.C., Thomson J.D. (2006). Within-host dynamics of an intestinal pathogen of bumble bees. Parasitology.

[33] Ruiz-González M.X., Bryden J., Moret Y., Reber-Funk C., Schmid-Hempel P., Brown M.J.F. (2012). Dynamic transmission, host quality, and population structure in a multihost parasite of bumblebees. Evolution.

