# Supplementary material for: Network Centrality as an Indicator for Pollinator Parasite Transmission via Flowers
Source: Insects. 2020 Dec 8;11(12):872. doi: 10.3390/insects11120872 (PMC7762566; doi:10.3390/insects11120872)
Supplement: Supplementary file 1 [file insects-11-00872-s001.pdf]

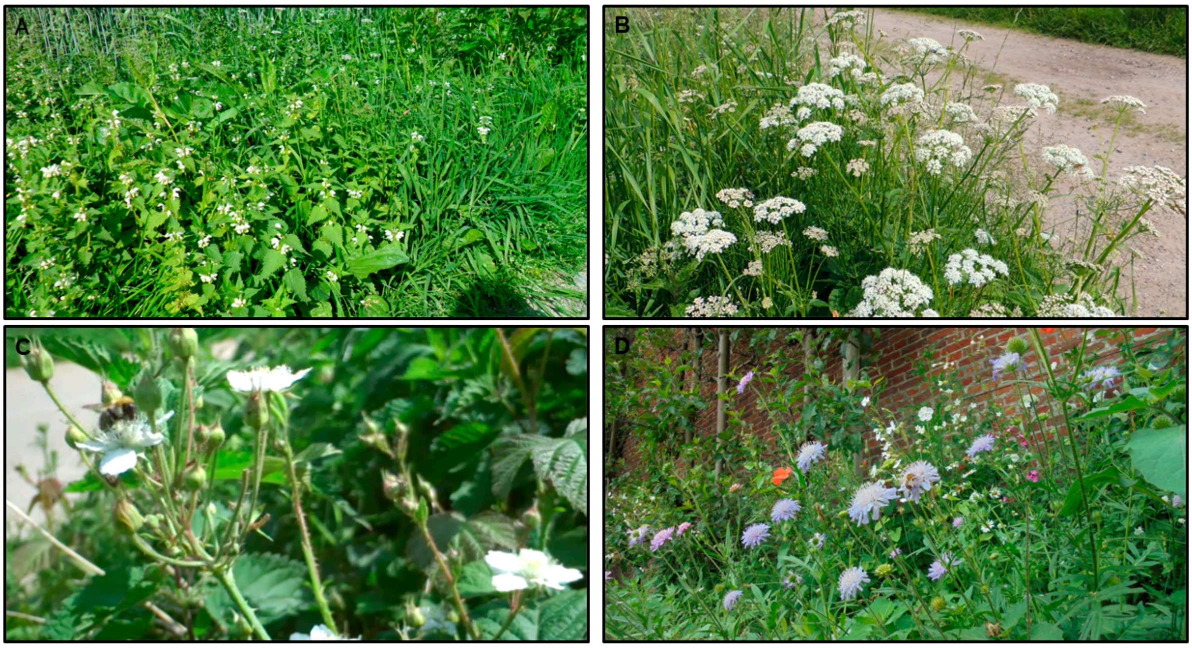

Figure S1. Pictures of the different study sites. (**A**) study site 1, *Lamium album* next to the wheat field. (**B**) study site 1, *Aegopodium podagraria* next to the dirt road. (**C**) study site 2, *B. pascuorum* on *Rubus fruticosus*. (**D**) study site 3, in the center of the photo: *B. pascuorum* on *Scabiosa coumbaria*.

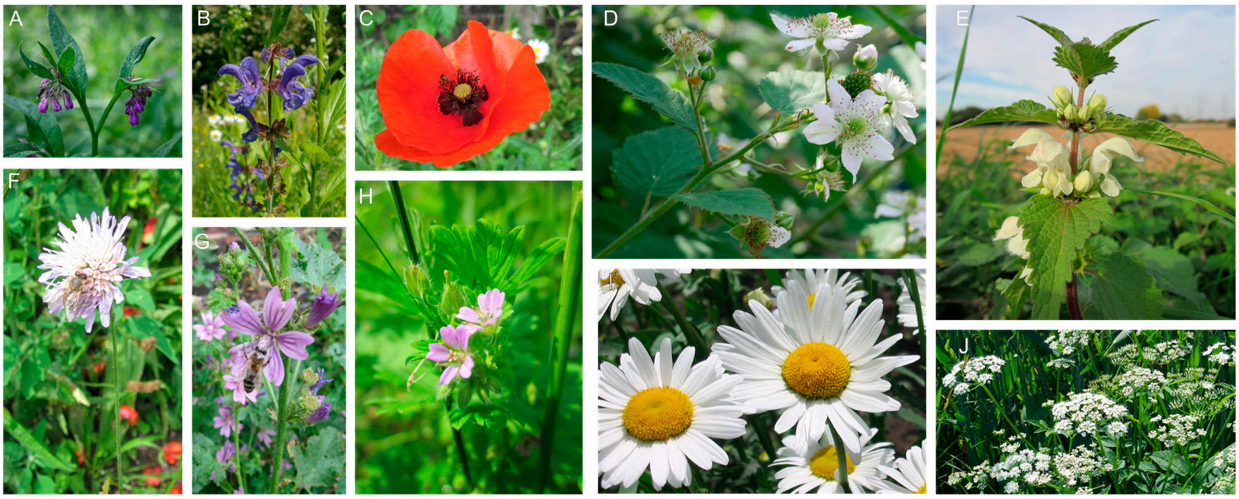

Figure S2. Pictures of the different flowers present in the 3 study sites, presence in the different study sites is indicated by the number between brackets. (**A**) *Symphytum officinale*{1–2}; (**B**) *Salva pratensis*{3}; (**C**) *Papaver rhoeas*{3}; (**D**) *Rubus fruticosus*{2}; (**E**) *Lamium album*{1–2}; (**F**) *Scabiosa columbaria*{3}; (**G**) *Malva neglecta*{3}; (**H**) *Geranium pusillum*{1}; (**I**) *Leucanthemum vulgare*{3}; (**J**) *Aegopodium podagraria*{1–2}. All plants in study site 1 and 2 are wild plants and common native species in Belgium. *S. officinale* is a perennial plant with white or purple tube-shaped flowers, it has a flower period May to August. *L. album* is a perennial plant and flowers from April to October. It has white flowers produced in verticillasters. *A. podagraria* is a perennial plant which flowers from June to July. It has small white open flowers which are organized in umbels. *G. pusillum* is an annual plant with small purple flowers, and flowers from May to October. *Rubus fruticosus* is a bush from the Rosaceae family with white or pink flowers, flowering from June to August [1]. The flowers in study site 3 were planted, yet, all species are endogenous to Belgium. *Salvia pratensis* is a perennial plant with different flower colors ranging from violet to pink and white; all plants in study site 3 had violet flowers. The flower period ranges from May to July. *Leucanthemum vulgare* has composite white-yellow flowers, which are open from May to August. *Papaver rhoeas* is an annual plant with red flowers, flowering from May to July. *Scabiosa columbaria* has blue-purple flowers and flowers from July to September. *Malva neglecta* is a perennial plant with white or blue-purple flowers, all flowers on Site 3 were purple, and it flowers from June to September [2].

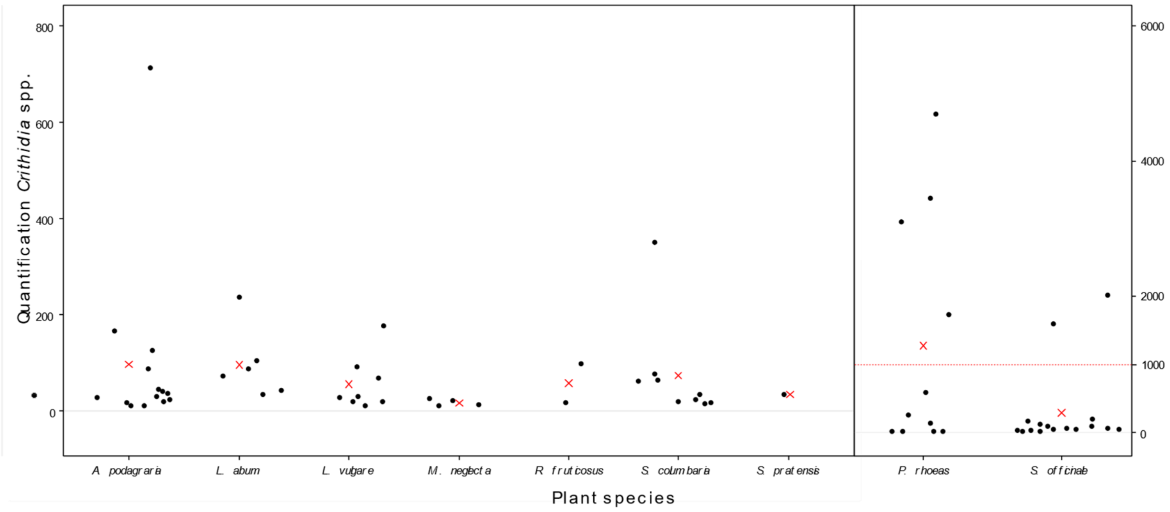

Figure S3. Dot plot of the quantification of *Crithidia* spp. on the flowers (only positive flowers are plotted) per plant species across all study sites. Red × denotes the mean quantity found on a plant species (only taking into account the positive flowers). Red dotted line represents the minimal infection dose if all infective particles are taken up, based upon values reported in literature, i.e. ca. 1000 infective cells [3]. Note the different scale for *P. rhoeas* and *S. officinale*. See also Table S4 for detailed quantification per plant species.

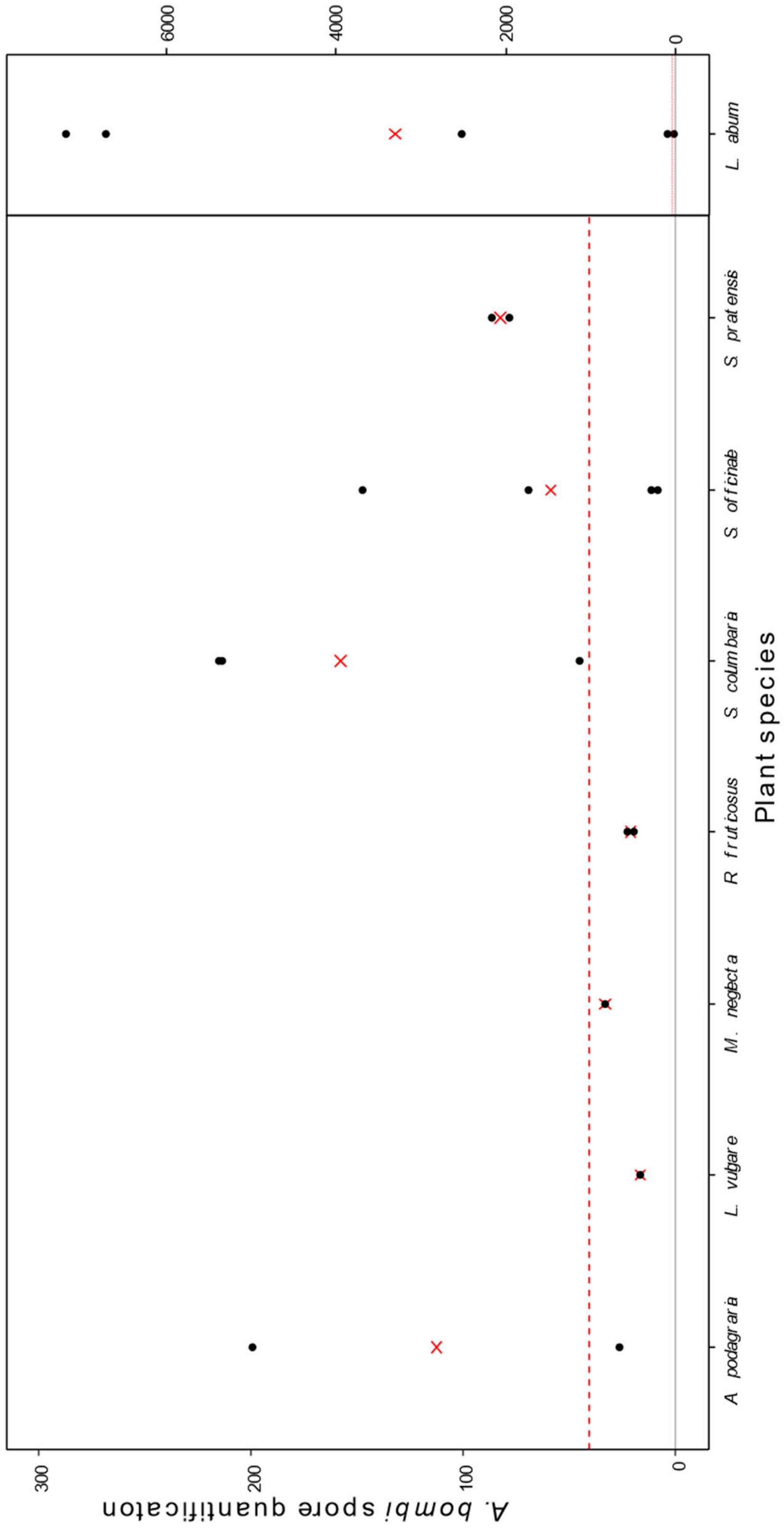

Figure S4. Dot plot of the quantification of *A. bombi* on the flowers (only positive flowers are plotted) per plant species across all study sites. Red × denotes the mean quantity found on a plant species (only taking into account the positive flowers). Red dotted line represents the minimal infection dose if all infective particles are taken up, based upon values reported in literature, i.e. ca. 40 oocysts [4]. Note the different scale for *L. album*. See also Table S 5 for detailed quantification per plant species.

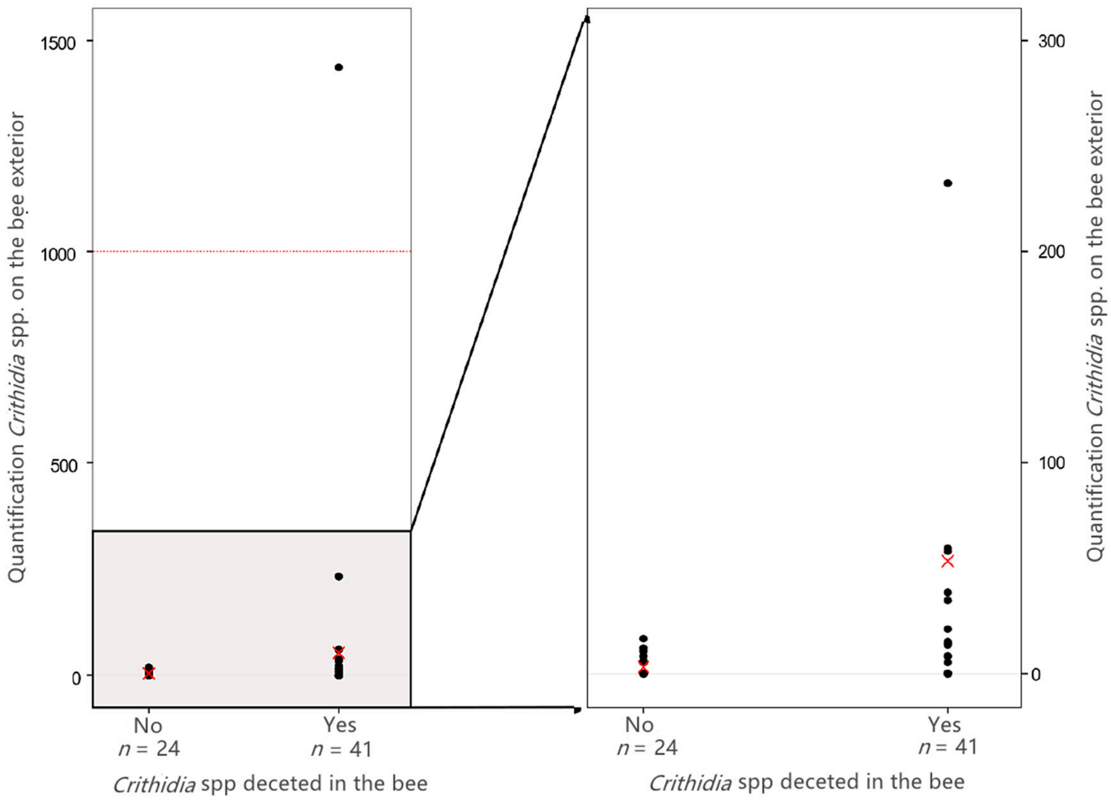

Figure S5. Dot plot of the quantification of *Crithidia* spp. found on the exterior of the bees across sites (i.e. all analyzed bees see Table S6 for more detail) (y-axis) the presence of *Crithidia* spp. within the bee is denoted on the x-axis. Right panel is an enlargement of the shaded rectangular on the left panel. Red × denotes the mean quantity found on the exterior of the bees. Red dotted line on the left panel represents the minimal infection dose if all infective particles are taken up, based upon values reported in literature, i.e. ca. 1000 infective cells [3].

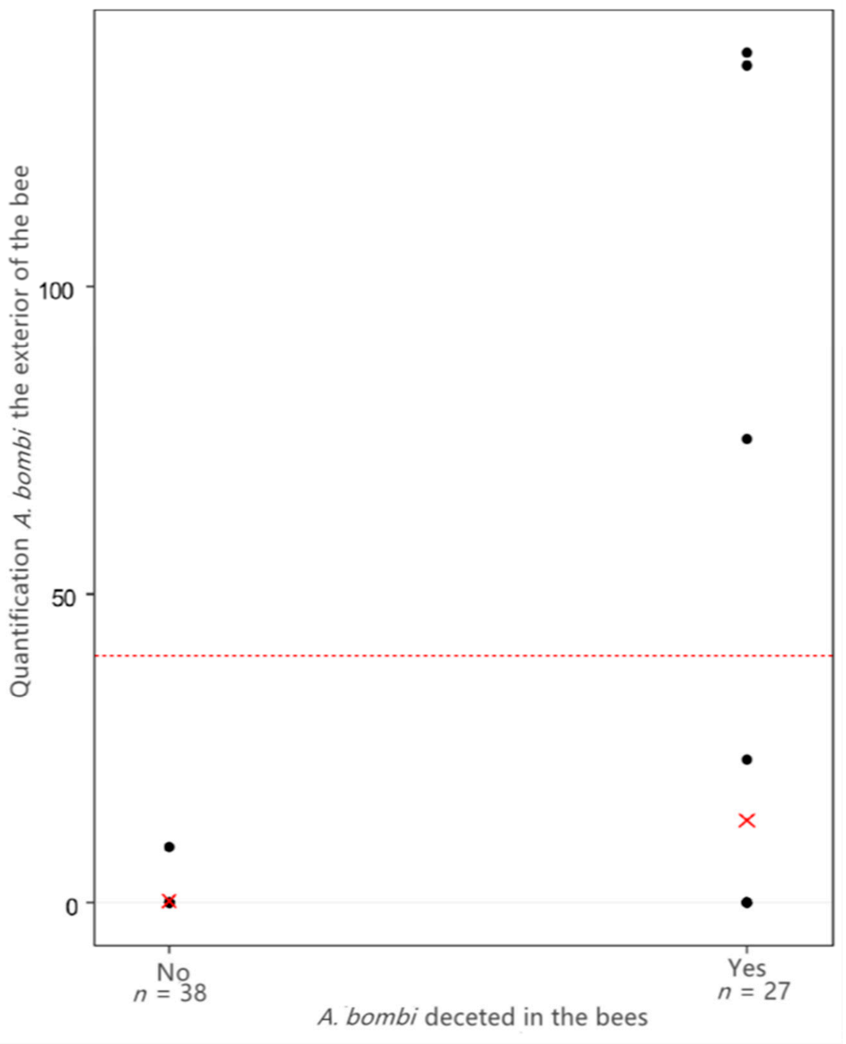

Figure S6. Dot plot of the quantification of *A. bombi* found on the exterior of the bees across sites (i.e. all analyzed bees see Table S6 for more detail) (y-axis) the presence of *A. bombi*. within the bee is denoted on the x-axis. Red × denotes the mean quantity found on the exterior of the bees. Red dotted line represents the minimal infection dose if all infective particles are taken up, based upon values reported in literature, i.e. ca. 40 oocysts [4].

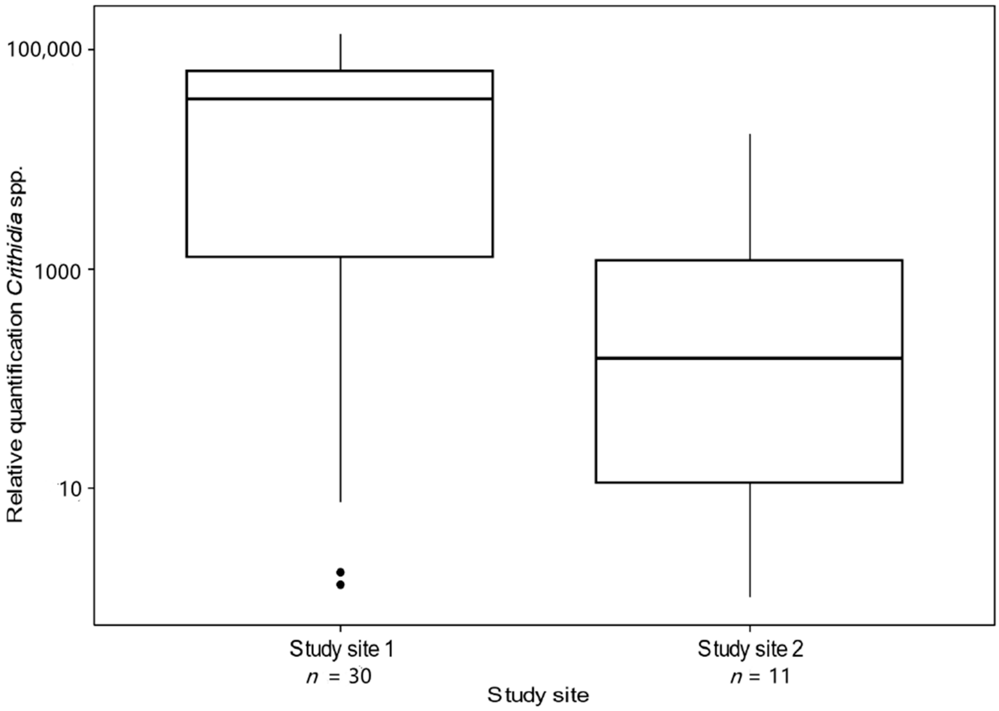

Figure S7. Boxplot of the Relative quantification of *Crithidia* spp. found in the bees per study site.

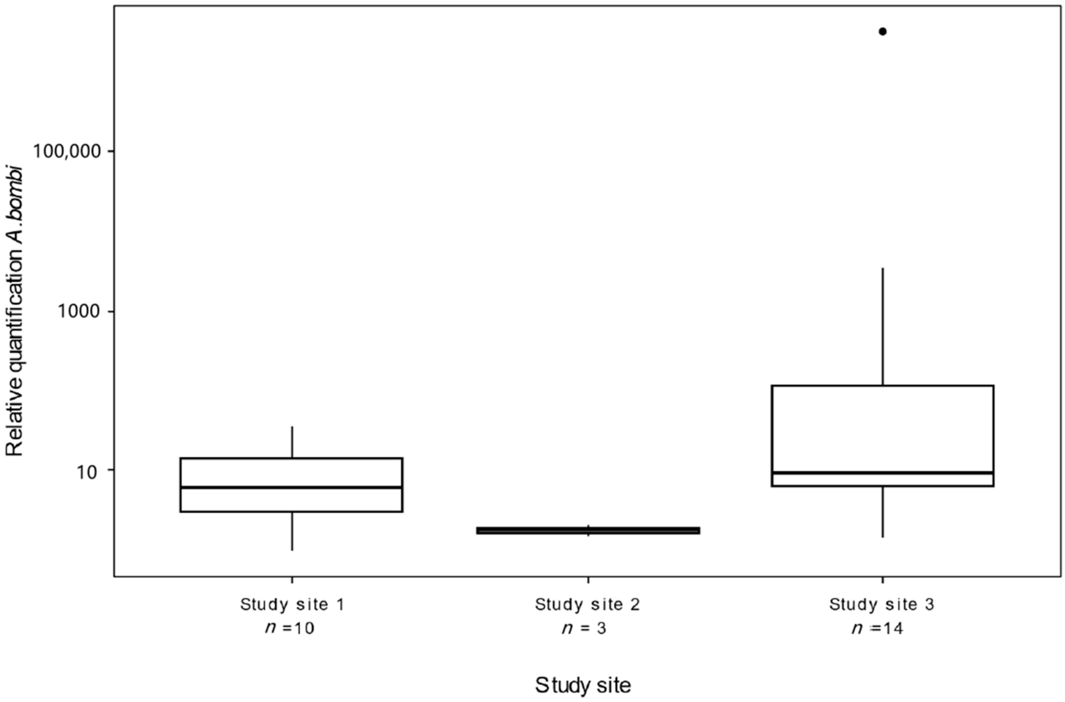

Figure S8. Boxplot of the Relative quantification of *A.bombi* found in the bees per study site.

Table S1. Flower species presence and abundance per species at the study sites. Species in bold were included in the network. The other species were not visited by any pollinator during 30 min monitoring and were therefore not included in the network.

**Study site 1**	
**Species**	**Counts**
Lamium album	198 flowers
Aegopodium podagraria	271 umbels
Symphytum officinale	221 flowers
Geranium pusillum	56 flowers
Taraxacum officinale	4 flowers
Tripleurospermum inodorum	57 flowers
Medicago lupulina	9 flower heads
Argentina anserina	81 flowers
Ranunculus acris	84 flowers
Achillea millefolium	22 umbels
**Study site 2**	
**Species**	**Counts**
Symphytum officinale	250 flowers
Aegopodium podagraria	41 umbels
Lamium album	104 flowers
Rubus fruticosus	36 flowers
**Study site 3**	
**Species**	**Counts**
Papaver rhoeas	18 flowers
Leucanthemum vulgare	215 flowers
Scabiosa columbaria	120 flowers
Malva neglecta	204 flowers
Salvia pratensis	83 flowers

Table S2. Pollinator counts from the performed transects at each study site. Dipteran species were only determined up to genus level. Transects were done by walking twice alongside the whole length of the study site and recording the pollinators present on flowers by visual inspection. These transects were not included in the network construction and were only used to assess the overall presence and activity of pollinators at each site. This to set up a monitoring plan for the camera recordings (see section 2.2. Network construction in the main text).

**Study site 1**	
**Species**	**Count**
Bombus terrestris	19
Bombus pascuorum	21
Bombus lapidarius	6
Apis mellifera	2
Lucilia sp.	28
Eristalis sp.	3
**Study site 2**	
**Species**	**Count**
Bombus pascuorum	14
Bombus pratorum	1
Bombus lapidarius	1
**Study site3**	
**Species**	**Count**
Bombus pascuorum	5
Bombus terrestris	2
Bombus lapidarius	1
Heriades truncorum	12
Apis mellifera	2
Eristalis sp.	3

Table S3. An overview of the number of sampled flowers for parasite analysis at each study site. Only the flower heads were sampled, and flowers were cut off in the field; for composite flowers such as *A. podagraria*, the whole umbel was sampled.

Study site 1	
**Species**	**Sampled**
Lamium album	24
Aegopodium podagraria	24
Symphytum officinale	24
Geranium pusillum	10
**Study site 2**	
**Species**	**Sampled**
Symphytum officinale	25
Aegopodium podagraria	23
Lamium album	25
Rubus fruticosus	23
**Study site 3**	
**Species**	**Sampled**
Papaver rhoeas	12
Leucanthemum vulgare	22
Scabiosa columbaria	20
Malva neglecta	21
Salvia pratensis	20

Table S4. An overview of the quantification of *Crithidia* spp. of the positive flowers per study site, qPCR quantification was based upon a standard curve (E = 87.2%; R^2 = 0.991 from a serial dilution of an in vitro culture of *C. mellificae* counted with a hemocytometer before DNA extraction).

**Study site**	**Plant species**	**Sample**	**Quantification *Crithidia* spp.**	**Study site**	**Plant species**	**Sample**	**Quantification *Crithidia* spp.**
1	*L. album*	D17	235	2	*A. podagraria*	LaZ5	27
1	*S. officinale*	Sp10	2020	2	*A. podagraria*	LaZ6	36
1	*S. officinale*	Sp9	1594	2	*A. podagraria*	LaZ8	44
1	*A. podagraria*	Z22	712	3	*S. columbaria*	GeDu1	18
2	*R. fruticosus*	LaB10	18	3	*S. columbaria*	GeDu10	77
2	*R. fruticosus*	LaB17	98	3	*S. columbaria*	GeDu11	24
2	*L. album*	LaD10	74	3	*S. columbaria*	GeDu12	65
2	*L. album*	LaD15	33	3	*S. columbaria*	GeDu13	18
2	*L. album*	LaD2	44	3	*S. columbaria*	GeDu3	62
2	*L. album*	LaD3	104	3	*S. columbaria*	GeDu4	15
2	*L. album*	LaD5	86	3	*S. columbaria*	GeDu7	349
2	*S. officinale*	LaS1	15	3	*S. columbaria*	GeDu9	33
2	*S. officinale*	LaS10	62	3	*M. neglecta*	GeKa1	21
2	*S. officinale*	LaS14	86	3	*M. neglecta*	GeKa10	9
2	*S. officinale*	LaS16	24	3	*M. neglecta*	GeKa14	12
2	*S. officinale*	LaS17	33	3	*M. neglecta*	GeKa6	24
2	*S. officinale*	LaS19	15	3	*P. rhoeas*	GeKl1	4701
2	*S. officinale*	LaS2	189	3	*P. rhoeas*	GeKl10	12
2	*S. officinale*	LaS20	59	3	*P. rhoeas*	GeKl11	254
2	*S. officinale*	LaS24	18	3	*P. rhoeas*	GeKl2	3450
2	*S. officinale*	LaS25	115	3	*P. rhoeas*	GeKl3	580
2	*S. officinale*	LaS3	160	3	*P. rhoeas*	GeKl4	6
2	*S. officinale*	LaS4	41	3	*P. rhoeas*	GeKl5	3110
2	*S. officinale*	LaS7	83	3	*P. rhoeas*	GeKl6	8
2	*S. officinale*	LaS8	47	3	*P. rhoeas*	GeKl7	1730
2	*A. podagraria*	LaZ1	166	3	*P. rhoeas*	GeKl8	127
2	*A. podagraria*	LaZ11	18	3	*P. rhoeas*	GeKl9	11
2	*A. podagraria*	LaZ13	86	3	*L. vulgare*	GeMA1	92
2	*A. podagraria*	LaZ14	18	3	*L. vulgare*	GeMA10	12
2	*A. podagraria*	LaZ17	12	3	*L. vulgare*	GeMA18	68
2	*A. podagraria*	LaZ2	24	3	*L. vulgare*	GeMA19	18
2	*A. podagraria*	LaZ20	127	3	*L. vulgare*	GeMA2	27
2	*A. podagraria*	LaZ21	30	3	*L. vulgare*	GeMA20	30
2	*A. podagraria*	LaZ3	41	3	*L. vulgare*	GeMA22	21
2	*A. podagraria*	LaZ4	12	3	*L. vulgare*	GeMA8	175
				3	*S. pratensis*	GeVS6	33

Table S5. An overview of the quantification of *Apicystis bombi* of the positive flowers per study site, qPCR quantification was based upon a standard curve (E = 91.4%; R^2 = 0.992 from a serial dilution of oocysts isolated from the fat body of a *B. pascuorum* worker, which were counted with a hemocytometer before DNA extraction).

Study site	Plant species	Sample	Quantification *A. bombi*
1	*L. album*	D10	7186
1	*L. album*	D2	6713
1	*L. album*	D13	2523
1	*S. officinale*	Sp3	147
1	*L. album*	D21	110
2	*A. podagraria*	LaZ11	199
2	*S. officinale*	LaS19	69
2	*A. podagraria*	LaZ3	26
2	*L. album*	LaD16	23
2	*R. fruticosus*	LaB23	22
2	*R. fruticosus*	LaB17	19
2	*S. officinale*	LaS20	11
2	*S. officinale*	LaS18	8
3	*S. columbaria*	GeDU16	215
3	*S. columbaria*	GeDU8	213
3	*S. pratensis*	GeVS6	86
3	*S. pratensis*	GeVS19	78
3	*S. columbaria*	GeDU15	45
3	*M. neglecta*	GeKa18	33
3	*L. vulgare*	GeMa16	16

Table S6. An overview of the number of sampled bee species at each study site and the number of *C. bombi* and *A. bombi* positive individuals (i.e. parasites were detected internally). Only the most prevalent bee species was sampled for parasite analysis. As in study site 1 the three Bombus species had a similar abundance, all three species were sampled.

**Study site 1**			
**Species**	**Sampled**	***C. bombi* positive**	***A. bombi* positive**
Bombus terrestris	12	11	3
Bombus pascuorum	12	10	5
Bombus lapidarius	10	9	2
**Study site 2**			
**Species**	**Sampled**	***C. bombi* positive**	***A. bombi* positive**
Bombus pascuorum	16	11	3
**Study site 3**			
**Species**	**Sampled**	***C. bombi* positive**	***A. bombi* positive**
Heriades truncorum	15	0	14

Table S7. Results of the GLMM (using Model 2, see main text) for the separate parasites. The presence/absence data of *A. bombi* or *Crithidia spp*. (both confirmed by Sanger sequencing) served as a binomial response variable for which the link function log of the odds ratio (logit) was used. The natural logarithm of the normalized weighted closeness was used as a fixed variable. Site and flower species served as random variable, where flower species was nested within site.

	Crithidia spp.			
**Fixed effect**	**β**	**df**	**□2**	**p-value**
Normalised closeness	4.458	4	7.852	0.005
	Apicystis bombi			
**Fixed effect**	**β**	**df**	**□2**	**p-value**
Normalised closeness	0.8816	4	0.835	0.361

References

1 H. Eggeltje and D. Tande Lid, *Veldgids Nederlandse flora*, 9e ed. Zeist: KNNV uitgeverij, 2015.

2 W. Van Landuyt, L. Vanhecke, and D. Brosens, "Florabank1: a grid-based database on vascular plant distribution in the northern part of Belgium (Flanders and the Brussels Capital region)," *PhytoKeys*, vol. 12, pp. 59–67, May 2012, doi: 10.3897/phytokeys.12.2849.

3 M. C. Otterstatter and J. D. Thomson, "Does pathogen spillover from commercially reared bumble bees threaten wild pollinators?," *PLoS One*, vol. 3, no. 7, p. e2771, Jan. 2008, doi: 10.1371/journal.pone.0002771.

4 P. Graystock, K. Yates, S. Evison, B. Darvill, D. Goulson, and W. O. H. Hughes, "The Trojan hives: pollinator pathogens, imported and distributed in bumblebee colonies," *J. Appl. Ecol.*, vol. 50, no. 5, p. n/a-n/a, Jul. 2013, doi: 10.1111/1365-2664.12134.
